# Photoaging Skin Therapy with PRP and ADSC: A Comparative Study

**DOI:** 10.1155/2020/2032359

**Published:** 2020-07-16

**Authors:** Luiz Charles-de-Sá, Natale Gontijo-de-Amorim, Andrea Sbarbati, Donatella Benati, Paolo Bernardi, Radovan Borojevic, Rosana Bizon Vieira Carias, Gino Rigotti

**Affiliations:** ^1^Postgraduate Program in Surgical Science, Federal University of Rio de Janeiro-CCS-Bloco C, Avenida Carlos Chagas Filho, 373, Ilha do Fundão, Rio de Janeiro, RJ 21941-902, Brazil; ^2^Dipartamento di Scienze Neurologiche e del Movimento, Sezione di Anatomia e Istologia della Universitá degli Studi di Verona, Strada Le Grazie 8, Verona 37134, Italy; ^3^Universidade Federal do Rio de Janeiro-UFRJ-CCS and Centro de Biotecnologia-IMETRO, Rio de Janeiro, Brazil; ^4^Casa di Cura San Francesco-Unità di Chirurgia Rigenerativa, Via Monte Ortigara, 21, Verona, Italy

## Abstract

**Background:**

Stem cells from adipose tissue (ADSCs) and platelet-rich plasma (PRP) are innovative modalities that arise due to their regenerative potential.

**Objective:**

The aim of this study was to characterize possible histological changes induced by PRP and ADSC therapies in photoaged skin.

**Methods:**

A prospective randomized study involving 20 healthy individuals, showing skin aging. They underwent two therapeutic protocols (protocol 1: PRP; protocol 2: ADSCs). Biopsies were obtained before and after treatment (4 months).

**Results:**

PRP protocol showed unwanted changes in the reticular dermis, mainly due to the deposition of a horizontal layer of collagen (fibrosis) and elastic fibers tightly linked. Structural analyses revealed infiltration of mononuclear cells and depot of fibrotic material in the reticular dermis. The ADSC protocol leads to neoelastogenesis with increase of tropoelastin and fibrillin. There was an improvement of solar elastosis inducing an increment of macrophage polarization and matrix proteinases. These last effects are probably related to the increase of elastinolysis and the remodeling of the dermis.

**Conclusions:**

The PRP promoted an inflammatory process with an increase of reticular dermis thickness with a fibrotic aspect. On the other hand, ADSC therapy is a promising modality with an important antiaging effect on photoaged human skin.

## 1. Introduction

The aging skin is a complex biological phenomenon that is composed of intrinsic and extrinsic processes. Facial aging is a degenerative process that affects the skin and deep structures resulting from the action of the intrinsic factor (age) associated with an extrinsic factor related to exposure to ultraviolet radiation [1–5]. The connective tissue of the skin is mainly composed of collagen and elastin. Collagen represents 70% to 80% of the dry weight of the skin, conferring mechanical strength and structural integrity. Elastic fiber is a minor component of the dermis, responsible for skin elasticity, with elastin protein representing 2% to 4% of the extracellular matrix [6]. In photoaging skin, there is progressive destruction of the entire dermal elastic fiber network, with thick, tangled, tortuous, degraded, and dysfunctional fibers, leading to the increased density of the elastic material, resulting in a cluster of amorphous and dystrophic elastotic material throughout the dermis, forming solar elastosis, the main feature of photoaging [6].

In recent years, there is an increasing interest in the use of the regenerative properties of adipose tissue [7, 8]. In this scope, some studies have suggested the application of fat grafting (lipotransferring) to improve skin quality [9, 10]. Animal model studies have described an improvement in skin texture and, in particular, increased elasticity, hyperpigmentation control, and aesthetic improvement of scars, regardless of etiopathogenesis [11–19].

Zuk et al.'s studies in 2001 [7, 20] identified mononucleated cells in the adipose tissue known as fibroblast-like cells with high capacity for cell differentiation. Mesenchymal stem cells, found in the stromal vascular fraction (SVF) of adipose tissue, are multipotent cells with high potential for self-renewal and *ex vivo* expansion, with the ability to differentiate into several cell lineages [21–26]. According to the International Society for Cell Therapy, minimum criteria are adopted to define a stem cell: characterization of MSC requires its phenotyping by identifying the cell surface membrane markers, its ability to adhere to the plastic surface, and the ability to differentiate into 2 to 3 distinct cell lineages [27].

Platelet-rich plasma (PRP) has also been described as having a regenerative effect due to growth factors and cytokines [28–31]. Currently, PRP therapy is described in many studies, as well as associated with other treatments [32–34]. PRP has been used to treat alopecia, acne, traumatic scars, contractile scars, wrinkles, stretch marks, chronic ulcers, laser therapy, and postsurgical wound to enhance the healing process. The PRP preparations have been used since the 1970s, and they have been increasingly popular since the 1990s [35]. Since then, different protocols and ways of preparing PRP have emerged: from conventional blood centrifugation to innovative systems. It can be activated by adding collagen, calcium, and/or thrombin, by glass contact or by freeze/thaw cycles, applied as a platelet or gel suspension [36–38].

At the moment, the biological mechanisms responsible for the rejuvenating and regenerative effect on human skin obtained with the fat graft and/or its components are not yet clearly understood, and there is no consensus on the histological and ultrastructural changes that occur in the skin and subcutaneous tissue, after ADSC and PRP therapies [39–43].

This study is aimed at evaluating the comparative histological changes of aged facial skin after subdermal therapy with PRP and ADSCs.

## 2. Patients, Materials, and Methods

This is a prospective clinical, randomized, and comparative study conducted between April 2013 and June 2017 with 20 healthy individuals (ASA 1: English, American Society of Anesthesiology) [44] of both genders, with ages ranging from 45 to 65 years, presenting photoaging skin variables, different skin color phototypes (Fitzpatrick) [45, 46], and cervicofacial sagging. The candidates who participated in this study were spontaneously submitted to the face-lifting surgical procedure at its end. All patients from northeastern Brazil were split into two skin therapy protocols: protocol 1 (PRP skin therapy) and protocol 2 (ADSC skin therapy).

The study was conducted at the Carlos Chagas Filho Institute of Biophysics, Federal University of Rio de Janeiro-Brazil, at the Excellion Services Biomedical SA® laboratory in Rio de Janeiro-Brazil, at the Center for Anatomy and Histology at the University of Verona-Italy and at Performa Ltda clinic, in Natal, RN, Brazil. All patients were recruited at the author's private clinic (Natal, RN), by electronic and/or verbal invitation and sent for clinical, cardiological, laboratory evaluation and signing of an informed consent form that was in accordance with the ethical principles of the Declaration of Helsinki 2000 and Istanbul 2008, following the norms of resolution 466/12 and subsequent ones of the National Health Council/Ministry of Health. It was submitted to the Ethics and Research Committee board to be approved (No. 28.063 at 05-25-2012) and registered in the Brazilian Registry of Clinical Trials (REBEC) on 04-07-2013 (RBR-2nn9y2).

Subjects with a smoking habit, with hematologic or hemodynamic disorders, autoimmune diseases, connective tissue diseases, diabetes type I or II, other metabolic diseases, and chronic use of corticosteroids, and those that had been submitted to recent dermatological or surgical face treatments (e.g., facial peeling) were not included. The direct endpoint of the study was to compare the histological benefits provided by the subdermal PRP and ADSC therapies.

### 2.1. Protocol 1: PRP Skin Therapy

#### 2.1.1. Blood Collection

Three 10 ml ACD tubes were collected with 3.2% sodium citrate solution (Vacutainer®, Ref: 369714; BD Biosciences). The three collected tubes containing blood were sent to the Excellion® laboratory. All the rules of transportation of human biological material were obeyed according to the resolution of the Federal Council of Medicine no. 1,823/2007. Tubes containing blood and 0.5 ml of 3.2% citrate solution were subjected to single-operator PRP preparation and stored at -20°C in a freezer.

#### 2.1.2. Preparation of Platelet-Rich Plasma (PRP)

For the development of the PRP, the protocol adopted by Amable et al. [28] was chosen. The collected hematic material was subjected to double centrifugation to ensure the best quality of the PRP, initially at 300 g for 5 minutes at 18°C and thereafter at 700 g for 17 minutes at 18°C. After the first centrifugation, all plasma above the clot layer (containing red and white cells (leukocytes)) was collected and named PRP1. This PRP1 was centrifuged at 700 g for 17 minutes at 18°C. After this second centrifugation step, the platelet pellet was resuspended in 300 *μ*l of poor platelet plasma (PPP), constituting a new fraction called PRP2. Platelet activation was performed by adding 20 mM CaCl_2_ (PRP2-Ca) (PRP2-Thr, Ref: T6884; Sigma-Aldrich, St. Louis, Missouri, USA), incubating at 37°C for 1 hour. For PRP2 activation, the samples were exposed to CaCl_2_ and centrifuged at 3,000 g for 20 minutes at 18°C. After activation of platelet growth factors, the solution was frozen at -20°C in a freezer. The day before application, the growth factor solution was thawed and kept refrigerated at 4°C. This PRP2 product represents a high platelet concentration (1 × 10^6^ platelets/*μ*l) 6.4 times higher than blood plasma. All steps were performed in a refrigerated centrifuge (certified Jouan Br4i, Saint-Herblain, Loire-Atlantique, France) [28].

#### 2.1.3. PRP Injection Procedure

After the preparation of the PRP (15-20 days), performed in the Excellion® laboratory, the PRP was placed in a 1 ml syringe (Luer Lock, BD®), coupled to the 30 G needle, containing a high concentration of platelets (1 × 10^6^ platelets/*μ*l). The application was performed in a subdermal plane in the left retroauricular skin in an area of 1 cm [2], 1 cm behind the auricular conchal.

### 2.2. Protocol 2: ADSC Skin Therapy

#### 2.2.1. Adipose-Derived Stem Cell (ADSC) Harvesting

Under local anesthesia with 0.5% xylocaine and 1,500,000 U epinephrine, 10 cc of adipose tissue from the infra-abdominal region was manually harvested by liposuction, using a 10 ml syringe (Luer Lock; BD®) coupled to a liposuction cannula of 3 mm diameter, 15 cm long, containing 3 distal holes (Tulip®, USA), with manual vacuum. The lipoaspirate was transferred to a glass vial containing RPMI culture medium and antibiotics (amphotericin and ciprofloxacin). It was transported under refrigeration to the laboratory to be processed accordingly to the specific protocol to isolate and expand ADSC.

#### 2.2.2. Isolation, Expansion, Characterization, and Differentiation of Adipose Mesenchymal-Derived Stem Cells (ADSCs)

Following the protocol originally described by Zuk et al. [7, 20], a 10 ml sample of the lipoaspirate was weighed after washing in sterile phosphate-buffered saline (PBS), pH 7.4, dissociated with collagenase IA (Sigma-Aldrich, USA), 200 U/mg of tissue, and incubated at 37°C under constant agitation for 1 h. The material was then centrifuged, and the pellet was filtered through a nylon mesh of 70 *μ*m. The cells were resuspended in culture medium supplemented with 10% fetal bovine serum (FBS), quantified using Trypan Blue, and plated in the low-glucose Dulbecco's medium (DMEM-Low, LGC) supplemented with 20% FBS and antibiotics (100 U/ml penicillin, 100 *μ*g/ml streptomycin). The following day (one passage), nonadherent cells were removed, and adherent cells were expanded in culture bottles.

Two days before application, the cells were washed with physiological saline and incubated in the culture medium supplemented with autologous plasma at 37°C under 5% CO_2_ to remove the potentially retained compounds of the bovine serum. One day before application, 1 ml Luer Lock®-type syringes were prepared containing each 2 million cells, diluted in 0.4 ml of physiological saline, and transported to the ambulatory facilities for injection.

ADSCs were characterized using combinations of surface markers of the mesenchymal cells, pericytes, and fibroblasts: CD105, CD90, CD73, CD14, CD45, CD34, and HLA-DR. The functional characterization of the cultured ADSCs was done after induction of differentiation in vitro to the osteogenic and adipogenic lineages following Zuk et al. protocol [7, 20].

#### 2.2.3. ADSC Injection Procedure

The expanded ADSCs were conditioned in 1 ml syringes attached to 30 G needle, containing 2 × 10^6^ cells in 0.4 ml PBS. The ADSCs were injected into the patient's subdermal skin, in an area of 1 cm [2] in the left preauricular region, 1 cm distally forward the tragus.

### 2.3. Skin Biopsies

Two pretreatment excisional skin biopsies (control biopsy = untreated skin) of the left retroauricular (3-5 mm posterior to the retroauricular groove) and preauricular regions (at 3-5 mm anterior to the tragus) were performed under local anesthesia with xylocaine 0.5% and epinephrine 1,500,000 U. Biopsies of a PRP- and ADSC-treated skin were taken after a 3-4 months of interval, during the procedure of face lifting. All the skin specimens were split into two parts. One part was submitted to paraffin embedding, and 4 *μ*m microtome slices were stained with hematoxylin-eosin (HE) and orcein with previous oxone oxidation to detect neoformed elastic fibers, as well as to immunohistochemistry. Another part was sent to the Center for Anatomy and Histology at University of Verona-Italy for ultrastructural analysis by scanning and transmission electron microscopy (SEM and TEM).

### 2.4. Immunohistochemistry

Paraffin sections were obtained and submitted to immunohistochemical techniques. The following antibodies were used: anti-tropoelastin (rabbit polyclonal, PR398, Elastin Products Co., Owensville, Missouri, USA), anti-fibrillin-1 (rabbit polyclonal, PR217, Elastin Products), anti-elastin (rabbit polyclonal, ab21607, Abcam, Cambridge, USA), anti-metalloproteinase 12 (MMP12) (rabbit monoclonal, Abcam), anti-cathepsin K (CAT-K) (mouse monoclonal, clone EP1261Y, ab52897, Abcam), anti-CD68 (monoclonal mouse, clone KPi, Dako, Carpinteria, USA), anti-mannose receptor (rabbit polyclonal, 64693, Abcam), and heme-oxigenase-1 (rabbit polyclonal, ab13243, Abcam). All reactions were performed using positive controls (breast cancer for MMP12 and kidney biopsy sections for CAT-K) and negative controls (instead of primary antibody, incubation with the same immunoglobulin isotype).

### 2.5. Histomorphometry

High-resolution images (2048 × 1536 pixels) were captured with a system of image analysis consisting of a digital photographic machine (Evolution VR Cooled Color 13 bits, Media Cybernetics®, Bethesda, USA) coupled to a light microscope (Eclipse E800, Nikon®, Japan). For the capture of images, the software Q-Capture 2.95.0, release 2.0.5 (Silicon Graphics Inc., EUA), was used. After program settings and calibration of color and contrast for each type of stain, the Image-Pro Plus 4.4.1 software (Media Cybernetics, Bethesda, MD 2002, USA) was used for quantification of either the stained or the immune-stained slices.

### 2.6. Statistical Analyses

Data were analyzed using SAS 6.112 (SAS Institute, Inc., Cary, NC) and expressed as medians (interquartile range). The Wilcoxon Rank Sum Test was used. An overall *α* level of 0.05 was used as the limit of statistical significance.

## 3. Results

### 3.1. Patients' Follow-Up

Twenty healthy subjects (4 males and 16 females) that participated in the present study were followed up regularly for four months after the subdermal PRP and ADSC injection until the skin removal has been done during the face-lifting surgery, and qualitative information regarding local skin reactions was analyzed. Local transitory effects were occasionally observed immediately after the cell injection, such as injection-site erythema, edema, bruising, or induration. They did not require any pharmaceutical intervention and returned to the reasonable skin condition within less than 48 h. No abnormal skin reactions were observed, such as persisting inflammation, necrosis, hyperplasic or abnormal cell growth, tumor development, or any other adverse events such as vasculitis or hypertrophic scars.

### 3.2. Preskin Therapy

**Figure 1:**
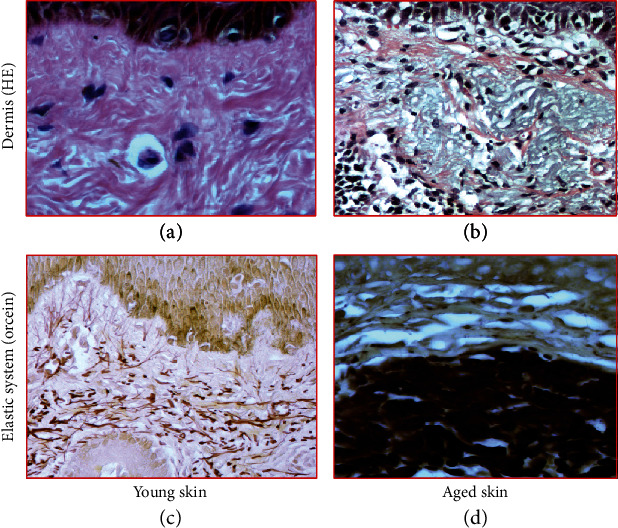
Histomorphological study of the skin. Skin biopsies: stained by (a, b) hematoxylin-eosin (HE) and (c, d) orcein: with previous oxidation with oxone to detect new elastic fibers. In (a), histological sections representative of young skin showing an organized arrangement of the structural components of the dermis with few inflammatory cells. In (b), intense inflammatory infiltrate and presence of degenerated material in the dermis, characteristic of collagen basophilic degeneration. In (c), microphotography of the elastic system: oxytalan fibers and elaunin fibers perpendicular to the DEJ; mature elastic fibers positioned horizontally in the deep dermis in a young skin. In (d), reduction of oxytalan fibers in zone 1 and accumulation of reactive material for oxidized orcein in the papillary and reticular dermis (actinic elastosis, zone 2). Original magnification: 4x (objective lens).

**Figure 2:**
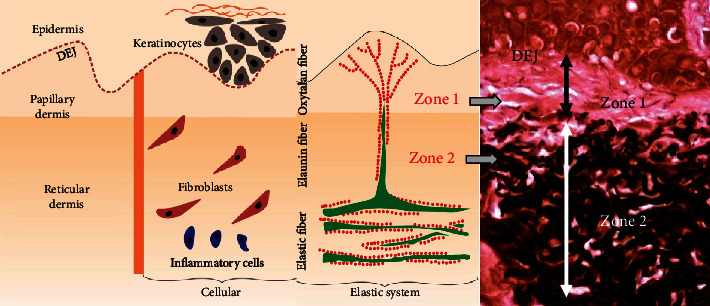
Definition of zones 1 and 2 of the dermis (40x magnification). Zone 1 is defined as an area delimited between the dermoepidermal junction (DEJ) and the presence of elastic material deposited in the deep dermis. Another band (zone 2) has been established which comprises the limits of the elastic material deposited more deeply in the dermis.

**Figure 3:**
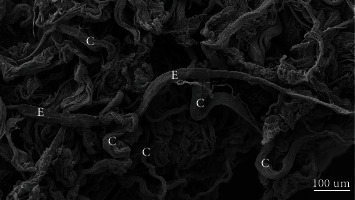
Photoaging of the skin. Representative photomicrograph of scanning electron microscopy (SEM): presence of a net of collagen fibers grouped in a disordered form (c) permeated by a large elastic mature fiber (E).

The pretreated skin showed different photoaging pattern of cutaneous elastosis, between the two regions/protocols. Due to the lower sun exposure in the left retroauricular region, the structural examination by scanning electron microscopy (SEM), the dermis showed few deposits of elastic and collagen fibers, which were scattered irregularly and intertwined in a network of collagen fibers, sometimes scattered in the reticular dermis without forming a common coalescent pattern of the elastosis. On the other hand, the left preauricular skin presented a solar elastosis, consequent to skin photoaging due to chronic sun exposure. In the papillary dermis (zone 1), a decrease of oxytalan and elaunin elastic network was observed, and in deep dermis (zone 2), the solar elastosis resulted in accumulation of the dense pathologic elastin and collagen fibers. These crumbled elastotic deposits can be directly related to the full loss of the hierarchically ordered network of elastin fibers, and their structure is not compatible with a normal elastic response to skin Stresses, leading to an overall loss of the skin elasticity in both zones (Figures 1 and 2). This is, in general, expected among the inhabitants of the NE coast of Brazil. The skin showed a consistent overall photoaged pathology, albeit it was present at different degrees of intensity (Figures 1(b) and 1(d)). In the structural examination by scanning electron microscopy (SEM), the dermis showed large diameter elastic fibers, irregularly dispersed and intertwined in a network of collagen fibers, sometimes condensed in the deeper plane of the dermis. The latter appeared rather scarce in some areas, but in others formed dense bundles (Figure 3). The histology showed a flattened dermal-epidermal junction (DEJ), with a reduction or a full loss of the dermal papillae. The major dermal structure involved in photoaging was the elastin component of the dermal extracellular matrix.

### 3.3. Post PRP Skin Therapy (Protocol 1)

Posttreatment biopsy of the patients showed the following findings.

**Figure 4:**
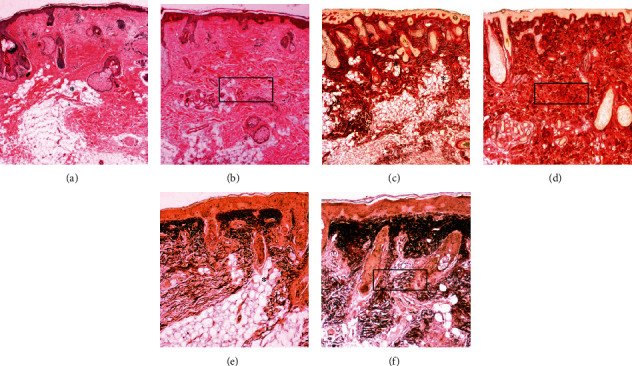
Histological analysis. Skin treatment with PRP: optical microscopy ((a, b) HE stain; (c, d) Picro-Sirius red stain; (e, f) oxidized orcein stain). (a, c, e) Pretreatment and (b, d, f) posttreatment using the PRP biopsies. In the pretreatment specimens, adipocytes are located at the boundary of the dermis with the subcutaneous tissue organized in lobules, which are directed vertically toward the surface of the skin (∗). These lobules form *adipose papillae*, which in their apical portion show a relationship with sebaceous or sweat glands. After the use of PRP, fibrosis is apparent through the thickening of the reticular dermis due to elastic fibers and collagen arranged horizontal and strongly attached (squares).

Treatment with PRP induced changes mainly in the reticular dermis. After the use of PRP, an increase in reticular dermis thickness was observed, which resulted in the horizontal deposition of layers of elastic fibers and collagen. In this layer, the presence of active fibroblasts was also observed. The significant increase in elastic and collagen fibers and the presence of fibroblasts suggest a fibrotic reaction in some areas. After the use of PRP, the relationship between subcutaneous fat and sebaceous or sweat glands appears less close due to the thickening of the reticular dermis developing a barrier between these elements (Figure 4).

**Figure 5:**
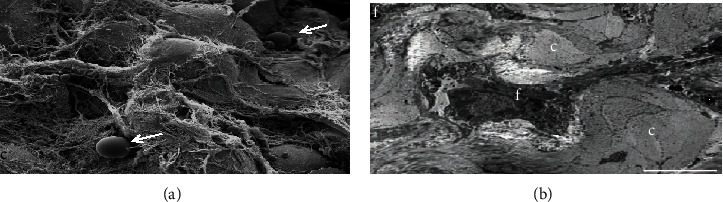
Scanning electron microscopy of the skin. Structural analysis of the deep layer of the dermis: (a) SEM shows an intense collagen network and inflammatory cells in reticular dermis (white arrows); (b) MET shows the presence of numerous fibroblasts in reticular dermis and collagen fibers in formation. f: *fibroblast*; c: *collagen*.

In structural analysis of posttreatment SEM biopsies (protocol 1: PRP), perivascular areas with mononuclear cell infiltration are visible in the reticular dermis and the dermoepidermal junction. The structural examination also confirmed the histological findings, revealing a large amount of elastic fibers and collagen mainly arranged parallel to the skin surface in compact bundles, as well as revealed the presence of fibroblasts with a large amount of rough endoplasmic reticulum and large lysosomes. These findings suggest that PRP stimulated the fibrosis process in the area where it was injected (Figure 5).

### 3.4. Post ADSC Skin Therapy (Protocol 2)

**Figure 6:**
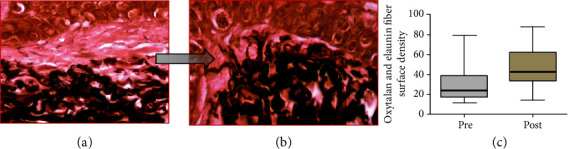
Photomicrographs representative of histological sections of biopsies before and after treatment with ADSCs. Changes in the pattern of zone 1 in copies (a) and (b) (oxidized orcein). (a) Pretreatment biopsy showing rare oxytalan fibers in zone 1. (b) Posttreatment biopsy shows a high density of perpendicular thin oxytalan fiber web stained: oxidized orcein. (c) Graphical representation of the surface density of oxytalan fibers in zone 1 (*p* = 0.0001).

All the biopsies of facial sun-aged skin submitted to subdermal injection of autologous ADSC showed different degrees of improvement of the overall skin structure, with partial or extensive reversal of the pathological signs typical of solar elastosis. In the zone 1 dermis, devoid of oxytalan and elaunin elastic network in the solar elastosis samples, a fully organized new system was found after the treatment. The regularly spaced oxytalan fiber bundles were crossing perpendicularly the region under the DEJ, linking it to the subjacent elastic fiber diffuse network laid parallel to the skin surface, indicating indeed an intense neoelastinogenesis in the regeneration of the solar elastosis (Figures 6(a)–6(c)). Histopathological analysis showed that the elastin component of the extracellular matrix of the skin was apparently the principal target of the therapy.

**Figure 7:**
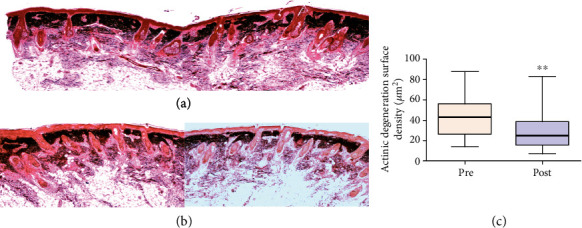
Photomicrographs representative of histological sections of biopsies before and after treatment with ADSCs. (a) Pretreatment biopsies showing intense actinic elastosis in the papillary and reticular dermis. (b) After treatment, a reduction in the elastotic mass in the dermis was observed. Stained: oxidized orcein. (c) Graph of the surface density of the reactive material for oxidized orcein in zone 2 reveals a significant reduction in elastosis (*p* = 0.005).

After the treatment, significant or full removal of the elastotic material was observed, associated with regeneration of the new sizeable elastic fiber network, with the structural organization compatible with the normal elasticity of the dermis (Figures 7(a)–7(c)).

**Figure 8:**
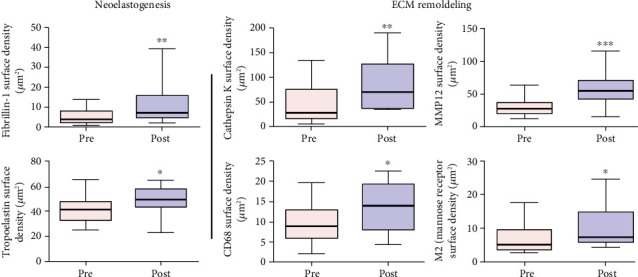
Immunohistochemical analysis after ADSC skin therapy into two aspects: neoelastogenesis represented by anti-fibrillin immune labelling that showed overall increase of fibrillin, including in the zone 1 (*p* = 0.001), and anti-tropoelastin immune labelling that showed increase of the tropoelastin reactive material in the post treatment biopsy (*p* < 0.05). Extracellular matrix (ECM) remodeling was represented by anti-cathepsin K immunostaining that revealed an increase in quantity of CAT-K immune-labelled cells in the dermis of a posttreated skin biopsy (*p* = 0.011), anti-MMP12 immunostaining representation of percentage of MMP12 in the skin biopsies significantly increased after ADSC treatment (*p* = 0.005), anti-CD68 and CD206 (mannose receptor (MR)) macrophage immunostaining in sun-exposed facial skin before and after ADSC injection that showed a significant increase after ADSC treatment in the dermis of posttreated skin biopsy (*p* < 0.05).

In the deep dermis, both fibrillin and tropoelastin were also slightly increased after the treatment when the morphology of the immune-labeled elastin molecules shifted from amorphous and crumbled elastotic deposits to the typical fibrillary structures (Figure 8).

Analysis of the total immune reactivity of cathepsin K and MMP12 showed a significant increase (*p* = 0.011 and *p* = 0.005, respectively) after treatment with ADSCs, which could justify the improvement in the elastosis pattern found in posttreatment skin (Figure 8).

It is notable that after ADSC therapy of sun-aged skin, the reactivation of cathepsin K expression in dermal fibroblasts or in infiltrating cells is observed. This activity required a second group of enzymes potentially involved in the elastinolysis observed in our mode which were the metalloproteinases, the major one being potentially the macrophage metalloelastase MMP12 [47, 48]. Similar to cathepsin K, the total intensity of the MMP12 immune labeling was significantly increased after the ADSC treatment of the sun-aged skin (Figure 8). The activities of the two enzymes were thus potentially complementary.

Classical macrophage activation upregulates several matrix MMPs, but the increase of the MMP12 mRNA levels is mainly associated with the alternative macrophage activation in the M2 phenotype [49]. All MSC are known to display an anti-inflammatory effect in the receptor tissues, due in large part to the induction of monocyte differentiation into M2 macrophages [50, 51]. Quantification of three markers of the M2 macrophage phenotype, CD68, CD206 (mannose receptor (MR)), and heme-oxygenase-1 (HO-1), is shown in Figure 8. All three showed a significant increase in positive cells following the ADSC treatment.

## 4. Discussion

Skin degenerative changes are related to age, hormonal changes, exposure to environmental agents, and stress [1–5]. In dermatology and plastic surgery, PRP has been widely applied for skin rejuvenation, scalp alopecia, associated with fat grafts, after laser peeling, and for prevention of hematoma in facelift procedures [52–56]. Derived growth factor platelets have been described for regenerative purposes [57] and other studies suggest paracrine effect, angiogenic action, and cell proliferation inducer [58, 59]. Despite the widespread use of PRP as a regenerative agent, its efficacy has yet to be confirmed in different clinical protocols, mainly for effects, the durability of results, proper dosing, preparation techniques, and comparison of results [35].

In a previous study, the effect of PRP in association with fat grafting was evaluated through a clinical trial. More pronounced inflammatory infiltrates and higher vascular reactivity were observed, with increased vascular permeability and a particular reactivity of nerve structures. The association of PRP with the graft did not produce the expected regenerative effect and had no advantages over the use of expanded ADSCs or SVF-enriched lipograft in skin rejuvenation [60]. However, PRP continues to be described as having a possible regenerative effect in other protocols due to their growth factors and cytokines [61–66].

Considering our results with isolated use of PRP in the skin, we observed that treatment with PRP promoted unwanted changes in the reticular dermis, mainly due to the deposition of a horizontal layer of collagen (fibrosis) and elastic fibers tightly linked creating a type of barrier between sweat and sebaceous glands with subcutaneous adipose tissue at the dermoepidermal junction [67]. Structural analyses after treatment with PRP showed infiltration of the perivascular area with mononuclear cells, visible in the reticular dermis and dermoepidermal junction. These areas showed blood capillaries and arterioles surrounding mononuclear infiltrates (Figure 5). In the skin treatment protocol using PRP, significant regenerative aspects in histological and ultrastructural analysis were not found [67–70]. Other aspects must be considered such as the half-life of platelet growth factors after activation (4-6 h), which may require several applications; and the standardization of terminologies and protocols to produce PRP, as well as the complete characterization of the final product, is fundamental for the comparability of results [28, 29].

Considering the PRP preparation method adopted in our trial [28], studies on cytokines have focused on the growth factors such as TGF, PDGF, VEGF, EGF, bFGF, HGF, IGF-1, PDGF, TGF, and EGF. Mazzocca et al. reported epidermal growth factor (EGF) which was higher than that in our protocol [71]. On the other hand, a similar platelet-derived growth factor (PDGF-AB) concentration (27.4 to 48.8 pg/10 [6] platelets) and a slightly lower transforming growth factor (TGF-*β*1) secretion (161.7 to 185.4 pg/10 [6] platelets) were reported [46]. Other authors reported similar values for TGF-*β*1/10 [6] platelets [72–74]. Castillo et al. related similar finding regarding PDGF-AB and PDGF-BB [75]. Differences in PRP preparation emphasize the necessity of standardization of methods, in order to make the comparative clinical analyses suitable.

The endpoint of the study was to compare the histological benefits provided by the subdermal PRP and ADSC therapies in photoaging skin. Due to the morphofunctional impairment of elastic fibers during the skin aging process, several therapeutic models have been used to maintain or replace elastin and elastic fiber levels [76, 77]. Treatments to repair and/or regenerate elastic fibers should consider all molecules involved in elastin and microfibril formation. Elastin constitutes over 90% of mature elastic fiber and is the main target of the recommended treatments. The use of tretinoin or retinoic acid in the skin in topical formulations has, for many years, increased elastin production by increasing tropoelastine [6, 77, 78] and fibrillin expression [79]. However, the biggest challenge is overcoming low-level tropoelastin expression in adult skin, meaning that such treatments are more likely to have only additional benefits on skin elastin density [80], neglecting the role of microfibrils (fibrillin) as a support structure for neosynthesized elastin deposition for forming a new elastic fiber. The microfibrils would adopt the structural function of a scaffold to allow the deposition of the newly formed elastin. Some mechanisms regulate the production and degradation of elastic fiber in adults through cytokines and signalers (TGF-*β*1, TGF-*α*, IGF-1, bFGF, EGF, TNF-a, and IL) by action on gene expression and tropoelastin transcription [81–93].

In ADSC skin therapy protocol, all analyses of the elastic system were directed at the reduction or total reversal of solar elastosis, addressing two issues: [1] regeneration of loss of oxytalanic and elaunin fiber networks in the papillary dermis and [2] replacement of pathological deposits of actinic elastin with a normal fibrillary structure, elastic fibers in the deep dermis. This remodeling action of the ECM at the elastic system level was demonstrated by the increased fiber density of the smaller diameter elastic system at the expense of new fibers in zone 1 (under DEJ). The density increase of this zone probably occurred due to the emergence of new oxytalanic/elaunin fibers shown in Figure 6. This pattern of neoelastogenesis was confirmed by the significant increase in fibrillin and tropoelastin (Figure 8) in ADSC posttreatment biopsies. Fibrillin is the main protein that makes up the microfibrils of oxytalan fiber, and its induction of expression precedes that of collagen. For this reason, it is considered a useful biomarker in skin repair processes. On the other hand, the degradation of the deposited elastic component (elastosis) was observed in the reticular dermis (zone 2) (Figure 7). Matrix metalloproteinase type 12 (MMP12) and cathepsin K (lysosomal elastase) are essential regulators of elastin degradation and are directly involved in extracellular matrix remodeling and elastotic material degradation processes [79, 94–99]. Once activated, cathepsin would not be able to degrade large polymerized elastic fibers, depending on the release of other ECM enzymes. It requires a group of complementary enzymes involved in elastinolysis (metalloproteinases), with MMP12 as its main representative and dependent on macrophage action [95, 96]. The mechanisms involved in the cellular degradation of cutaneous elastosis related to the presence of type M2 macrophages were analyzed using M2 markers (CD 206: receptor mannose), which showed a significant increase in skin biopsies after treatment with ADSCs.

In ADSC-treated skin, inflammatory cells may be implicated in the resolution of the ECM repair and reorganization process by polarizing the macrophage population to M2 [49]. These findings suggest a direct contribution of ADSCs to skin regeneration [42]. Macrophages regulate various matrix metalloproteinases (MMPs), but the increase in MMP12 mRNA levels is mainly associated with the alternative activation of macrophages with M2 phenotype [94]. All MSCs are known to have an anti-inflammatory effect on tissues due to largely inducing macrophage polarization to M2 [100–103]. In this study, M2 phenotype markers were monitored in dermal tissue samples that received treatment with ADSCs. Quantification of M2 macrophages by CD206 (mannose receptor (MR)) revealed the increase of this subpopulation in posttreatment biopsies. The anti-inflammatory effect mediated by MSC in recipient tissues is largely due to the increase in M2 macrophages in treated tissues [104], and we propose that this is at least in part one of the major regenerative activities of ADSCs in our study model.

Regarding the aspect of biosafety in tissue and cell transfer, despite the short time interval of the present study (4 months), between the application of ADSCs and their effects on tissue samples submitted to the action of ADSCs, no dysplastic or oncogenic changes in the skin of the population studied were observed as found in the literature [105]. The mesenchymal cells of the stromal fraction of adipose tissue used in this assay are autologous and homologous (ADSCs derived from adipose tissue were used elsewhere with the same histological and embryonic structure) [105–109].

The limitations of this study were the difficulty of accurately quantifying skin photoaging, morphofunctional analysis of all elements involved in the regenerative process after treatment with PRP and ADSCs, and knowledge of all involved biological events and their mechanisms.

The advantages of using isolated stem cells as antiaging skin therapy over PRP, fat grafting, and/or ADSC-matching therapies are as follows: there is no risk of cyst formation and fibrosis, use of small injection volumes with high regenerative potential, precise application, possibility of reapplication after reexpansion of stored stem cells, and/or cryopreserved cell reuse.

The future of this research line is aimed at creating new possibilities in regenerative therapy not only in skin diseases but in other clinical applications in the case of organs and tissues with reduction and/or alteration in the elastic system (e.g., aneurysms and joint problems), with a better understanding of the mechanisms involved and the control of these processes.

## 5. Conclusion

It was concluded that the action of PRP when injected on aged human skin induces an inflammatory process, contributing to the increase of collagen fiber deposits and the increase of reticular dermis thickness with a fibrotic aspect, not bringing any significant tissue regenerative role. On the other hand, expanded ADSC therapy in photoaged skin is related to ECM remodeling, increased production of new elastic fibers, and degradation of elastotic material deposited in the dermis (elastosis), inducing an important regenerative effect that could be considered a promising skin rejuvenation therapy.

## Data Availability

The data used to support the findings of this study are available from the corresponding author upon request.

## References

[1] Naylor E. C., Watson R. E. B., Sherratt M. J. (2011). Molecular aspects of skin ageing. *Maturitas*.

[2] Uitto J., Matsuoka L. Y., Kornberg R. L., Rudolph R. (1986). Elastic fibers in cutaneous elastoses. *Problems in Aesthetic Surgery: Biological Causes and Clinical Solutions*.

[3] Scharffetter-Kochanek K., Brenneisen P., Wenk J. (2000). Photoaging of the skin from phenotype to mechanisms. *Experimental Gerontology*.

[4] Oikarenen A. (1990). The aging of skin: chronoaging versus photoaging. *Photodermatology Photoimmunology & Photomedicine*.

[5] Fisher G. J., Kang S., Varani J. (2002). Mechanisms of photoaging and chronological skin aging. *Archives of Dermatology*.

[6] Uitto J. (1979). Biochemistry of the elastic fibers in normal connective tissues and its alterations in diseases. *The Journal of Investigative Dermatology*.

[7] Zuk P. A., Zhu M., Mizuno H. (2001). Multilineage cells from human adipose tissue: implications for cell-based therapies. *Tissue Engineering*.

[8] Rigotti G., Marchi A., Sbarbati A. (2009). Adipose-derived mesenchymal stem cells: past, present, and future. *Aesthetic Plastic Surgery*.

[9] Guillaume-Jugnot P., Daumas A., Magalon J. (2016). State of the art. Autologous fat graft and adipose tissue-derived stromal vascular fraction injection for hand therapy in systemic sclerosis patients. *Current Research in Translational Medicine*.

[10] Coleman S. R. (2006). Structural fat grafting: more than a permanent filler. *Plastic and Reconstructive Surgery*.

[11] Lorenz H. P., Hedrick M. H., Chang J., Mehrara B. J., Longaker M. T. (2000). The impact of biomolecular medicine and tissue engineering on plastic surgery in the 21st century. *Plastic and Reconstructive Surgery*.

[12] Mojallal A., Lequeux C., Shipkov C. (2009). Improvement of skin quality after fat grafting: clinical observation and an animal study. *Plastic and Reconstructive Surgery*.

[13] Vecchio D. D., Rohrich R. J. (2012). A classification of clinical fat grafting. *Plastic and reconstructive surgery*.

[14] Coleman S. R. (1997). Facial recontouring with lipostructure. *Clinics in Plastic Surgery*.

[15] Corrêa W. E., Garofalo F., Pitanguy I. (1997). Treatment of Romberg’s disease with combination of aspirated fat graft Ptfe-E and Medpor. *Revista Brasileira de Cirurgia*.

[16] Coleman S. R., Katzel E. B. (2015). Fat Grafing for Facial Feeling. *Clin Plast Surg*.

[17] Gonzalez A. M., Lobocki C., Kelly C. P., Jackson I. T. (2007). An alternative method for harvest and processing fat grafts: an in vitro study of cell viability and survival. *Plastic and Reconstructive Surgery*.

[18] Gir P., Oni G., Brown S. A., Mojallal A., Rohrich R. J. (2012). Human adipose stem cells. *Plastic and Reconstructive Surgery*.

[19] Moseley T. A., Zhu M., Hedrick M. H. (2006). Adipose-Derived stem and progenitor cells as fillers in plastic and reconstructive surgery. *Plastic and Reconstructive Surgery*.

[20] Zuk P. A., Zhu M., Ashjian P. (2002). Human adipose tissue is a source of multipotent stem cells. *Molecular Biology of the Cell*.

[21] Eto H., Suga H., Matsumoto D. (2009). Characterization of structure and cellular components of aspirated and excised adipose tissue. *Plastic and Reconstructive Surgery*.

[22] Brown S. A., Levi B., Lequex C., Wong V. W., Mojallal A., Longaker M. T. (2010). Basic science review on adipose tissue for clinicians. *Plastic and Reconstructive Surgery*.

[23] Stosich M. S., Mao J. J. (2007). Adipose tissue engineering from human adult stem cells: clinical implications in plastic and reconstructive surgery. *Plastic and Reconstructive Surgery*.

[24] Rydén M., Dicker A., Götherström C. (2003). Functional characterization of human mesenchymal stem cell-derived adipocytes. *Biochemical and Biophysical Research Communications*.

[25] Rigotti G., Marchi A., Gali?? M. (2007). Clinical treatment of radiotherapy tissue damage by lipoaspirate transplant: a healing process mediated by adipose-derived adult stem cells. *Plastic and Reconstructive Surgery*.

[26] Li H., Zimmerlin L., Marra K. G., Donnenberg V. S., Donnenberg A. D., Rubin J. P. (2011). Adipogenic Potential of adipose stem cell subpopulations. *Plastic and Reconstructive Surgery*.

[27] Dominici M., Le Blanc K., Mueller I. (2006). Minimal criteria for defining multipotent mesenchymal stromal cells. The International Society for Cellular Therapy position statement. *Cytotherapy*.

[28] Amable P. R., Carias R. B., Teixeira M. V. (2013). Platelet-rich plasma preparation for regenerative medicine: optimization and quantification of cytokines and growth factors. *Stem Cell Research & Therapy*.

[29] Elghblawi E. (2018). Platelet-rich plasma, the ultimate secret for youthful skin elixir and hair growth triggering. *Journal of Cosmetic Dermatology*.

[30] Xiong B. J., Tan Q. W., Chen Y. J. (2018). The Effects of Platelet-Rich Plasma and Adipose-Derived Stem Cells on Neovascularization and Fat Graft Survival. *Aesthetic Plastic Surgery*.

[31] Smith O. J., Jell G., Mosahebi A. (2019). The use of fat grafting and platelet-rich plasma for wound healing: A review of the current evidence. *International Wound Journal*.

[32] Nita A. C., Orzan O. A., Filipescu M., Jianu D. (2013). Fat graft, laser CO₂ and platelet-rich-plasma synergy in scars treatment. *Journal of Medicine and Life*.

[33] Klosova H., Stetinsky J., Bryjova I., Hledik S., Klein L. (2013). Objective evaluation of the effect of autologous platelet concentrate on post-operative scarring in deep burns. *Burns*.

[34] Picard F., Hersant B., Niddam J., Meningaud J. P. (2017). Injections of platelet-rich plasma for androgenic alopecia: a systematic review. *Journal of Stomatology, Oral and Maxillofacial Surgery*.

[35] Marx R. E. (2001). Platelet-rich plasma (Prp): what is Prp and what is not Prp?. *Implant Dentistry*.

[36] Mazzucco L., Balbo V., Cattana E., Guaschino R., Borzini P. (2009). Not every PRP-gel is born equal. Evaluation of growth factor availability for tissues through four Prp-gel preparations: Fibrinet®, RegenPRP-Kit®, Plateltex® and one manual procedure. *Vox Sanguinis*.

[37] Kaux J.-F., Le Goff C., Seidel L. (2011). Comparative study of five techniques of preparation of platelet-rich plasma. *Pathologie Biologie*.

[38] Oudelaar B. W., Peerbooms J. C., Huis In 't Veld R., Vochteloo A. J. H. (2019). Concentrations of Blood Components in Commercial Platelet-Rich Plasma Separation Systems: A Review of the Literature. *The American Journal of Sports Medicine*.

[39] Fadadu P. P., Mazzola A. J., Hunter C. W., Davis T. T. (2019). Review of concentration yields in commercially available platelet-rich plasma (PRP) systems: a call for PRP standardization. *Regional Anesthesia & Pain Medicine*.

[40] Rigotti G., Charles-de-Sá L., Gontijo-de-Amorim N. F. (2016). Expanded stem cells, stromal-vascular fraction, and platelet-rich plasma enriched fat: comparing results of different facial rejuvenation approaches in a clinical trial. *Aesthetic Surgery Journal*.

[41] Bosset S., Barre P., Chalon A. (2002). Skin ageing: clinical and histopathologic study of permanent and reducible wrinkles. *European Journal of Dermatology*.

[42] Kim J. H., Jung M., Kim H. S., Kim Y. M., Choi E. H. (2011). Adipose-derived stem cells as a new therapeutic modality for ageing skin. *Experimental Dermatology*.

[43] Meruane M. A., Rojas M., Marcelain K. (2012). The use of adipose tissue-derived stem cells within a dermal substitute improves skin regeneration by increasing neoangiogenesis and collagen synthesis. *Plastic and Reconstructive Surgery*.

[44] Fitz-Henry J. (2011). The Asa classification and peri-operative risk. *Annals of the Royal College of Surgeons of England*.

[45] Fitzpatrick T. B. (1988). The validity and practicality of sun-reactive skin types I through Vi. *Archives of Dermatology*.

[46] Fitzpatrick R. E., Rostan E. F. (2009). Reversal of photodamage with topical growth factors: a pilot study. *Journal of Cosmetic and Laser Therapy*.

[47] Pellicoro A., Aucott R. L., Ramachandran P. (2012). Elastin accumulation is regulated at the level of degradation by macrophage metalloelastase (Mmp-12) during experimental liver fibrosis. *Hepatology*.

[48] Saarialho-Kere U., Kerkelä E., Jeskanen L. (1999). Accumulation of matrilysin (Mmp-7) and macrophage metalloelastase (Mmp 12) in actinic damage. *The Journal of Investigative Dermatology*.

[49] Mantovani A., Biswas S. K., Galdiero M. R., Sica A., Locati M. (2013). Macrophage plasticity and polarization in tissue repair and remodelling. *The Journal of Pathology*.

[50] Zhao H., Shang Q., Pan Z. (2018). Exosomes From Adipose-Derived Stem Cells Attenuate Adipose Inflammation and Obesity Through Polarizing M2 Macrophages and Beiging in White Adipose Tissue. *Diabetes*.

[51] Mahbub S., Deburghgraeve C. R., Kovacs E. J. (2012). Advanced age impairs macrophage polarization. *Journal of Interferon & Cytokine Research*.

[52] Frautschi R. S., Hashem A. M., Halasa B., Cakmakoglu C., Zins J. E. (2016). Current evidence for clinical efficacy of platelet rich plasma in aesthetic surgery: a systematic review. *Aesthetic Surgery Journal*.

[53] Lee J. W., Kim B. J., Kim M. N., Mun S. K. (2011). The efficacy of autologous platelet rich plasma combined with ablative carbon dioxide fractional resurfacing for acne scars: a simultaneous split-face trial. *Dermatologic Surgery*.

[54] Kamakura T., Kataoka J., Maeda K. (2015). Platelet-rich plasma with basic fibroblast growth factor for treatment of wrinkles and depressed areas of the skin. *Plastic and Reconstructive Surgery*.

[55] Lim H.-K., Suh D.-H., Lee S.-J., Shin M. K. (2014). Rejuvenation effects of hyaluronic acid injection on nasojugal groove: prospective randomized split face clinical controlled study. *Journal of Cosmetic and Laser Therapy*.

[56] Blanton M. W., Hadad I., Johnstone B. H. (2009). Adipose stromal cells and platelet-rich plasma therapies synergistically increase revascularization during wound healing. *Plastic and Reconstructive Surgery*.

[57] Nakamura S., Ishihara M., Takikawa M. (2010). Platelet-rich plasma (Prp) promotes survival of fat-grafts in rats. *Annals of Plastic Surgery*.

[58] James I. B., Coleman S. R., Rubin J. P. (2016). Fat, stem cells, and platelet-rich plasma. *Clinics in Plastic Surgery*.

[59] Fukaya Y., Kuroda M., Aoyagi Y. (2012). Platelet-rich plasma inhibits the apoptosis of highly adipogenic homogeneous preadipocytes in an in vitro culture system. *Experimental & Molecular Medicine*.

[60] Charles-de-Sá L., Gontijo-de-Amorim N. F., Takiya C. M. (2015). Antiaging treatment of the facial skin by fat graft and adipose-derived stem cells. *Plastic and Reconstructive Surgery*.

[61] Zhu S.-J., Choi B.-H., Jung J.-H. (2006). A comparative histologic analysis of tissue-engineered bone using platelet- rich plasma and platelet-enriched fibrin glue. *Oral Surgery, Oral Medicine, Oral Pathology, Oral Radiology, and Endodontics*.

[62] Cervelli V., Bocchini I., Di Pasquali C. (2013). P.R.L. Platelet Rich Lipotransfert: our experience and current state of art in the combined use of fat and PRP. *BioMed Research International*.

[63] Sommeling C. E., Heyneman A., Hoeksema H., Verbelen J., Stillaert F. B., Monstrey S. (2013). The use of platelet-rich plasma in plastic surgery: a systematic review. *Journal of Plastic, Reconstructive & Aesthetic Surgery*.

[64] Hirase T., Ruff E., Surani S., Ratnani I. (2018). Topical application of platelet-rich plasma for diabetic foot ulcers: a systematic review. *World Journal of Diabetes*.

[65] Kakudo N., Minakata T., Mitsui T., Kushida S., Notodihardjo F. Z., Kusumoto K. (2008). Proliferation-promoting effect of platelet-rich plasma on human adipose-derived stem cells and human dermal fibroblasts. *Plastic and Reconstructive Surgery*.

[66] Kawasumi M., Kitoh H., Siwicka K. A., Ishiguro N. (2008). The effect of the platelet concentration in platelet-rich plasma gel on the regeneration of bone. *The Journal of Bone and Joint Surgery British volume*.

[67] Charles-de-Sá L., Gontijo-de-Amorim N. F., Takiya C. M. (2018). Effect of use of platelet-rich plasma (Prp) in skin with intrinsic aging process. *Aesthetic Surgery Journal*.

[68] Lei X., Xu P., Cheng B. (2019). Problems and solutions for platelet-rich plasma in facial rejuvenation: a systematic review. *Aesthetic Plastic Surgery*.

[69] Li P., Zhang R., Zhou Q. (2017). Efficacy of platelet-rich plasma in retarding intervertebral disc degeneration: a meta-analysis of animal studies. *BioMed Research International*.

[70] Shi W. J., Tjoumakaris F. P., Lendner M., Freedman K. B. (2017). Biologic injections for osteoarthritis and articular cartilage damage: can we modify disease?. *The Physician and Sportsmedicine*.

[71] Mazzocca A. D., McCarthy M. B. R., Chowaniec D. M. (2012). Platelet-rich plasma differs according to preparation method and human variability. *The Journal of Bone and Joint Surgery American Volume*.

[72] Weibrich G., Kleis W. K. G., Hafner G., Hitzler W. E. (2002). Growth factor levels in platelet-rich plasma and correlations with donor age, sex, and platelet count. *Journal of Cranio-Maxillo-Facial Surgery*.

[73] Zimmermann R., Arnold D., Strasser E. (2003). Sample preparation technique and white cell content influence the detectable levels of growth factors in platelet concentrates. *Vox Sanguinis*.

[74] Sundman E. A., Cole B. J., Fortier L. A. (2011). Growth factor and catabolic cytokine concentrations are influenced by the cellular composition of platelet-rich plasma. *The American Journal of Sports Medicine*.

[75] Castillo T. N., Pouliot M. A., Kim H. J., Dragoo J. L. (2011). Comparison of growth factor and platelet concentration from commercial platelet-rich plasma separation systems. *The American Journal of Sports Medicine*.

[76] Braverman I. M., Fonferko E. (1982). Studies in cutaneous aging: I. The elastic fiber network. *The Journal of Investigative Dermatology*.

[77] Mecham R. P., Je H., Hay E. D. (1991). The elastic fibre. *Cell Biology Of Extracellular Matrix*.

[78] Tajima S., Hayashi A., Suzuki T. (1997). Elastin expression is up-regulated by retinoic acid but not by retinol in chick embryonic skin fibroblasts. *Journal of Dermatological Science*.

[79] Watson R. E. B., Long S. P., Bowden J. J., Bastrilles J. Y., Barton S. P., Griffiths C. E. M. (2008). Repair of photoaged dermal matrix by topical application of a cosmetic ‘antiageing’ product. *The British Journal of Dermatology*.

[80] Sephel G. C., Buckley A., Davidson J. M. (1987). Developmental initiation of elastin gene expression by human fetal skin fibroblasts. *The Journal of Investigative Dermatology*.

[81] Sproul E. P., Argraves W. S. (2013). A cytokine axis regulates elastin formation and degradation. *Matrix Biology*.

[82] Imayama S., Braverman I. M. (1989). A hypothetical explanation for the aging of skin. Chronologic alteration of the three-dimensional arrangement of collagen and elastic fibers in connective tissue. *The American Journal of Pathology*.

[83] Guay M., Lagace G., Lamy F. (2009). Photolysis and ozonolysis of desmosine and Elastolytic peptides. *Connective Tissue Research*.

[84] Tsuji T. (1987). Different effects of elastase on dermal elastic fibers with age. *Gerontology*.

[85] Sephel G. C., Davidson J. M. (1986). Elastin production in human skin fibroblast cultures and its decline with age. *Journal of Investigative Dermatology*.

[86] Robinet A. (2005). Elastin-derived peptides enhance angiogenesis by promoting endothelial cell migration and tubulogenesis through upregulation of Mt1-Mmp. *Journal of Cell Science*.

[87] Lambros V. (2009). Improvement of skin quality after fat grafting: clinical observation and an animal study. *Plastic and Reconstructive Surgery*.

[88] El-Domyati M., El-Ammawi T. S., Moawad O. (2012). Efficacy of mesotherapy in facial rejuvenation: a histological and immunohistochemical evaluation. *International Journal of Dermatology*.

[89] Fu X., Li H. (2009). Mesenchymal stem cells and skin wound repair and regeneration: possibilities and questions. *Cell and Tissue Research*.

[90] Chan R. K., Zamora D. O., Wrice N. L. (2012). Development of a vascularized skin construct using adipose-derived stem cells from debrided burned skin. *Stem Cells International*.

[91] Strem B. M., Hicok K. C., Zhu M. (2005). Multipotential differentiation of adipose tissue-derived stem cells. *The Keio Journal of Medicine*.

[92] Planat-Benard V., Silvestre J.-S., Cousin B. (2004). Plasticity of human adipose lineage cells toward endothelial cells: physiological and therapeutic perspectives. *Circulation*.

[93] Jurgens W. J. F. M., Oedayrajsingh-Varma M. J., Helder M. N. (2008). Effect of tissue-harvesting site on yield of stem cells derived from adipose tissue: implications for cell-based therapies. *Cell and Tissue Research*.

[94] Huang W.-C., Sala-Newby G. B., Susana A., Johnson J. L., Newby A. C. (2012). Classical macrophage activation up-regulates several matrix metalloproteinases through mitogen activated protein kinases and nuclear factor-*κ*B. *PLoS One*.

[95] Lagente V., Le Quement C., Boichot E. (2009). Macrophage metalloelastase (MMP-12) as a target for inflammatory respiratory diseases. *Expert Opinion on Therapeutic Targets*.

[96] Rünger T. M., Quintanilla-Dieck M. J., Bhawan J. (2007). Role of cathepsin K in the turnover of the dermal extracellular matrix during scar formation. *The Journal of Investigative Dermatology*.

[97] Codriansky K. A., Quintanilla-Dieck M. J., Gan S., Keady M., Bhawan J., Rünger T. M. (2009). Intracellular degradation of elastin by cathepsin K in skin fibroblasts – a possible role in photoaging. *Photochemistry and Photobiology*.

[98] Novinec M., Grass R. N., Stark W. J., Turk V., Baici A., Lenarčič B. (2007). Interaction between human cathepsins K, L, and S and elastins. *The Journal of Biological Chemistry*.

[99] Turk V., Stoka V., Vasiljeva O. (2012). Cysteine cathepsins: from structure, function and regulation to new frontiers. *Biochimica et Biophysica Acta (BBA) - Proteins and Proteomics*.

[100] Guo S., Wang T., Zhang S. (2020). Adipose-derived stem cell-conditioned medium protects fibroblasts at different senescent degrees from UVB irradiation damages. *Molecular and Cellular Biochemistry*.

[101] Taddese S., Weiss A. S., Neubert R. H. H., Schmelzer C. E. H. (2008). Mapping of macrophage elastase cleavage sites in insoluble human skin elastin. *Matrix Biology*.

[102] Li C., Xu M. M., Wang K., Adler A. J., Vella A. T., Zhou B. (2018). Macrophage polarization and meta-inflammation. *Transl. Res.*.

[103] Rinnerthaler M., Bischof J., Streubel M., Trost A., Richter K. (2015). Oxidative stress in aging human skin. *Biomolecules*.

[104] Zachar L., Bacenkova D., Rosocha J. (2016). Activation, homing, and role of the mesenchymal stem cells in the inflammatory environment. *Journal of Inflammation Research*.

[105] Laverdet B., Micallef L., Lebreton C. (2014). Utilisation des cellules souches mesenchymateuses pour la reparation cutanee et l'elaboration de substituts de peau. *Pathologie Biologie*.

[106] Barrientos S., Stojadinovic O., Golinko M. S., Brem H., Tomic-Canic M. (2008). Perspective article: growth factors and cytokines in wound healing. *Wound Repair and Regeneration*.

[107] Pérez-Cano R., Vranckx J. J., Lasso J. M. (2012). Prospective trial of adipose-derived regenerative cell (ADRC)-enriched fat grafting for partial mastectomy defects: the Restore-2 trial. *European Journal of Surgical Oncology*.

[108] Gontijo-de-Amorim N. F., Charles-de-Sá L., Rigotti G. (2017). Mechanical supplementation with the stromal vascular fraction yields improved volume retention in facial lipotransfer: a 1-year comparative study. *Aesthetic Surgery Journal*.

[109] Anderi R., Makdissy N., Azar A., Rizk F., Hamade A. (2018). Cellular therapy with human autologous adipose-derived adult cells of stromal vascular fraction for alopecia areata. *Stem Cell Research & Therapy*.

